# Improving drug response prediction via integrating gene relationships with deep learning

**DOI:** 10.1093/bib/bbae153

**Published:** 2024-04-09

**Authors:** Pengyong Li, Zhengxiang Jiang, Tianxiao Liu, Xinyu Liu, Hui Qiao, Xiaojun Yao

**Affiliations:** School of Computer Science and Technology,Xidian University, 710126 Xi’an, Shaanxi, China; State Key Laboratory of Quality Research in Chinese Medicine, Macau Institute for Applied Research in Medicine and Health, Macau University of Science and Technology, 519020 Macau, China; School of Electronic Engineering, Xidian University, 710126 Xi’an, Shaanxi, China; School of Computer Science and Technology,Xidian University, 710126 Xi’an, Shaanxi, China; Beijing Laboratory of Biomedical Materials, Department of Geriatric Dentistry, Peking University School and Hospital of Stomatology, 100081 Beijing, China; Department of Oncology, Tai’an Municipal Hospital, 271021 Tai’an, Shandong, China; Centre for Artificial Intelligence Driven Drug Discovery, Faculty of Applied Sciences, Macao Polytechnic University, 999078 Macao, China

**Keywords:** drug response, pharmacogenomics, deep learning

## Abstract

Predicting the drug response of cancer cell lines is crucial for advancing personalized cancer treatment, yet remains challenging due to tumor heterogeneity and individual diversity. In this study, we present a deep learning-based framework named Deep neural network Integrating Prior Knowledge (DIPK) (DIPK), which adopts self-supervised techniques to integrate multiple valuable information, including gene interaction relationships, gene expression profiles and molecular topologies, to enhance prediction accuracy and robustness. We demonstrated the superior performance of DIPK compared to existing methods on both known and novel cells and drugs, underscoring the importance of gene interaction relationships in drug response prediction. In addition, DIPK extends its applicability to single-cell RNA sequencing data, showcasing its capability for single-cell-level response prediction and cell identification. Further, we assess the applicability of DIPK on clinical data. DIPK accurately predicted a higher response to paclitaxel in the pathological complete response (pCR) group compared to the residual disease group, affirming the better response of the pCR group to the chemotherapy compound. We believe that the integration of DIPK into clinical decision-making processes has the potential to enhance individualized treatment strategies for cancer patients.

## Graphical Abstract

**Graphical Abstract:**
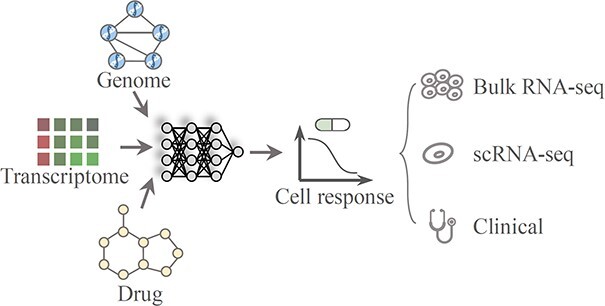

## INTRODUCTION

Human cancers often exhibit significant heterogeneity in virtually all discernible phenotypic features, which is a substantial barrier to successful personalized treatment [1, 2]. With the advent of genomic profiling technologies, pharmacogenomics [3–6] opens up new opportunities for personalized medicine development. Pharmacogenomics aims to study how genomic alterations and transcriptomic programming affect drug response, allowing for personalized drug treatment based on a patient’s unique genetic profile [7]. In recent years, high-throughput drug screening technologies have enabled researchers to conduct large-scale experiments on the drug response of cancer cell lines [8–10]. Projects such as Genomics of Drug Sensitivity in Cancer (GDSC) [11] and Cancer Cell Line Encyclopedia (CCLE) [12] have analyzed the genomic profiles of multiple cancer cell lines and drug sensitivity against these cell lines [13]. These pharmacogenomics datasets provide a more comprehensive representation of the genetic basis for variability in drug metabolism and efficacy [14, 15].

Recently, many deep learning (DL) models [16] have been developed utilizing pharmacogenomics datasets for predicting drug sensitivity, and have shown superior performance than traditional machine learning [17–24]. Generally, many of these methods take advantage of the powerful feature extraction ability of DL to learn the representations for cell lines and drugs, and then merge the learned representations to yield a final prediction. Diverse modeling strategies exist for cell lines and drugs. For example, Manica *et al*. [25] proposed an attention-based convolutional network to learn the representation of drug SMILES [26] and cell gene expression profiles in 2019. As it is computationally intractable to process the high-dimensional gene data, they screen out a subset of informative genes by network propagation over a protein-protein interaction network [27]. Jia *et al*. [28] introduced a deep variational autoencoder to compress gene expression into latent vectors in 2021 and demonstrated that these vectors can accurately predict drug response. Chawla *et al*. [29] demonstrate the benefits of considering drug descriptors and gene pathway features in 2022.

Although these initial studies have made great progress in drug response prediction, there is controversy over characterizing cells and drugs. Most methods use transcriptomic features (gene expression profiles) for cell line representation; however, there is growing evidence that drug responses could be modulated by the concerted behavior and complex interactions of multiple genes [30–34]. Thus, using only transcriptomic features without gene relationships might limit the accuracy and robustness of response prediction. Besides, current methods mainly use molecular fingerprints or SMILES to characterize the drug compounds that fail to encode the molecular topological information.

To address these issues, we presented the **D**eep neural network **I**ntegrating **P**rior **K**nowledge (DIPK) for cancer drug response prediction, a DL framework designed to incorporate the prior knowledge of gene interaction relationships, gene expression profiles and molecular topology in the prediction of cancer drug response. The extraction and integration of prior knowledge is efficiently handled through the application of self-supervised technologies and attention mechanisms. The self-supervised technologies employed in DIPK enable the model to learn meaningful representations from unlabeled data, leveraging the abundant information available in the gene and compound. This approach enhances the model’s ability to capture intricate patterns and relationships within the data, thereby improving prediction performance. By leveraging attention mechanisms, DIPK could effectively focus on relevant features and prioritize the importance of different components, further enhancing its predictive power. Through the incorporation of gene interaction relationships and molecular topology, DIPK captures the complex interplay between genes and drugs, providing a more comprehensive understanding of drug response mechanisms. This integration of prior knowledge allows DIPK to leverage existing biological knowledge and enhance its predictive capabilities.

To assess the predictive capabilities of DIPK, we conducted cross-validation on both GDSC and CCLE datasets to demonstrate the accuracy of DIPK in predicting drug response for known cell lines and drugs. Additionally, we examined the framework’s extrapolation ability by evaluating its performance on novel cell lines and drugs. The remarkably lower prediction errors obtained through our DIPK model indicate its superiority over state-of-the-art approaches in drug sensitivity prediction. Furthermore, DIPK showcases its robust generalizability by enabling accurate drug response prediction using single-cell expression profiles. This capability highlights the potential for DIPK to effectively handle diverse data types. Significantly, our study also demonstrates the reliability of DIPK in predicting drug response using patient data. By analyzing breast cancer patient datasets, DIPK successfully distinguished between patients with or without pathological complete response (pCR) [35]. This finding suggests the potential application of DIPK in clinical treatment settings. To our knowledge, this study is the first attempt to integrate the prior knowledge of gene interaction relationships and drug topology to model drug sensitivity. The exceptional performance of the DIPK framework across various datasets and its ability to handle different types of data further underscore its potential in advancing the development of personalized medicine.

## METHODS

### Model framework

**Figure 1:**
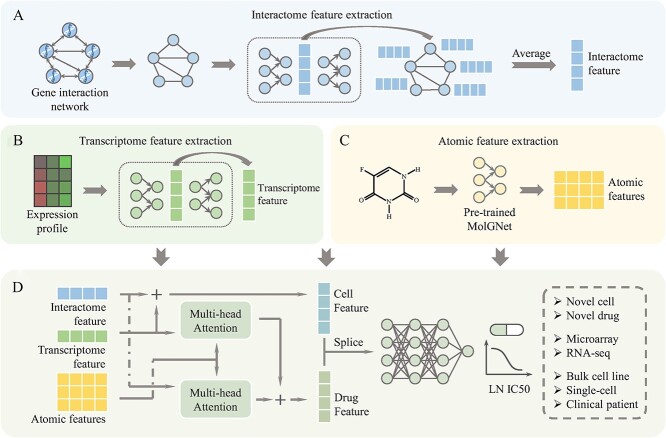
Model framework of DIPK. (**A**) The gene interaction network was encoded by GAE to obtain gene features that incorporate the interactions. The highly expressed gene features were averaged to represent the interactome feature of a given cell line. (**B**) The gene expression profile was compressed into a low-dimensional hidden vector using DAE. (**C**) The drug was represented by a molecular graph and then input into MolGNet to obtain the atomic features. (**D**) The interactome feature, transcriptome feature and atomic features were integrated using the multi-head attention layers and fully connected layers to get the LN IC50.

Accurate modeling of cells and drugs constitutes the cornerstone of drug response prediction. In particular, cells are characterized by their genetic information, which is encoded within the deoxyribonucleic acid present in each cell. The behavior and properties of a cell are profoundly influenced by gene interactions and gene expression patterns [36]. Therefore, to effectively encapsulate the intrinsic features of cells, DIPK adopts a robust biological network integration framework known as BIONIC [37]. BIONIC seamlessly integrates multiple high-quality gene interaction network s, culminating in the acquisition of a unified 512-dimensional vector through the utilization of a graph auto-encoder (GAE) [38]. This process involves aggregating highly expressed gene features derived from BIONIC to characterize intricate gene interaction relationships inherent to a given cell line (see Figure 1A). Additionally, DIPK employs a denoising auto-encoder (DAE) [39] to compress the gene expression profile of the cell line into a low-dimensional hidden vector (Figure 1B). By fusing the characterization of gene relationships with gene expression profiling, DIPK achieves a more holistic understanding of the cell. This integrated approach enhances the ability to capture the complex interplay between gene interactions and gene expression, providing a more accurate representation of the cell’s characteristics.

To characterize the drug compound, DIPK utilizes a pre-trained molecular graph neural network (MolGNet) [40], which performs the translation of the molecular graph representation into atomic continuous vectors through a neighbor attention-based message-passing approach (see Figure 1C). This pre-trained MolGNet is adept at capturing meaningful patterns within the molecular topological structure, leading to interpretable and expressive representations. As shown in Figure 1(D), DIPK employs a multi-head attention mechanism [41] to fuse the cell and drug information, generating a comprehensive drug representation. The drug representation is combined with the cell representation and then fed into the fully connected layer to generate the final drug response prediction. By incorporating both cell and drug information, DIPK provides a comprehensive framework for accurate drug response prediction. The integration of gene interaction relationships, gene expression profiling, and molecular topological structure enhances the understanding of complex cellular processes and drug mechanisms.

Overall, DIPK consists of Cell Modeling, Drug Modeling, Fusion and Output. Among them, representations of cells are comprised of interactome features and transcriptome features, and representations of drugs are molecular graphs. The model framework is described in detail below.

#### Cell modeling

As shown in Figure 1D, cell characterization consists of the interactome feature and transcriptome feature. For the interactome feature, we identified the top 256 genes with the highest expression levels. The 512-dimensional representations of these highly expressed genes were extracted with BIONIC [37], a comprehensive framework that combines multiple high-quality gene interaction networks using a GAE (Figure 1A). The average of these highly expressed gene representations is considered the interactome feature of the cell, encompassing valuable information about gene interactions. We compressed the gene expression profile into a 512-dimensional hidden vector that was considered the transcriptome feature. Specifically, we employed a DAE comprising a sequence of Rectified Linear Unit (ReLU) activated fully connected layers with dimensions [2048, 1024, 512, 1024, 2048] (Figure 1B) [39, 42]. During pre-training, the gene expression profile with random noise is input into DAE. The Mean Squared Error (MSE) between the original expression profile and the reconstructed one is treated as the loss function.

#### Drug modeling

To construct a molecular graph, the atomic and chemical bonding details of a drug compound molecule are retrieved through its SMILES string. Within the graph structure, we assigned atoms as nodes and bonds as edges. This graph is then employed as input for MolGNet, a framework that facilitates the generation of an encoded representation (Figure 1C) [40]. In this representation, each atom is represented by a vector of 768 dimensions. The MolGNet contains a message calculation function M and a vertex update function U, and these two components work as follows: 


(1) \begin{align*} & m_{i}^{t}=\mathrm{M}\left(\left\{x_{i}^{t-1}, x_{j}^{t-1}, e_{i j}\right\}, j \in \mathcal{N}_{i}\right) \end{align*}

(2) \begin{align*} & x_{i}^{t}=\mathrm{U}(h_{i}^{t-1}, m_{i}^{t}) \end{align*}

where $\mathcal{N}_{i}$ represents the neighbors of node $i$, $e_{i j}$ denotes the edge between the node $i$ and node $j$, vertex update function U is a gated recurrent unit network [43], $h_{i}^{t-1}$ is the hidden state of U and $h_{i}^{0}$ is the initial atom representation $x_{i}^{0}$. The MolGNet is pre-trained with both Pairwise Half-graph Discrimination [44], which is designed to discriminate whether two half-graphs are of the same source, and graph attribute masking strategy.

#### Fusion and output

Following the extraction of cell and drug representations, the fusion and output process integrates various features to generate the final output. The interactome feature and transcriptome feature are subjected to a similar sequence of operations, involving a ReLU-activated linear layer, followed by a separate multi-head attention layer that incorporates the atom features. The attention mechanism promotes a higher degree of interaction between these features, thus enhancing the representational capacity of the model. In the majority of cases, parameter sharing is implemented between corresponding layers in the interactome feature and transcriptome feature pipelines (i.e. between the two linear layers or between the two multi-head attention layers). This tactic is known to boost the model’s generalization ability by enabling it to identify and exploit commonalities between different types of input features. However, when the task of our method is to predict drug responses for learned cell lines and drugs, the need for generalization is less critical. Stemming from this kind of consideration, the parameters between these layers are kept distinct, allowing the model to focus on achieving more nuanced learning. The output of the attention layer is computed as follows: 


(3) \begin{align*}& Attention(Q, K, V)=softmax\left(\frac{Q K^{T}}{\sqrt{d_{k}}}\right) V\end{align*}

where *$d_{k}$* is the dimension of the atomic features, which are fed into two independent linear layers to obtain the matrices *K* and *V*. The encoded interactome feature or transcriptome feature is fed into a linear layer without the activation function to obtain the matrix *Q*. The molecular feature is derived by summing the outputs of the two multi-head attention layers. The interactome feature is then encoded by a ReLU-activated linear layer. This layer performs a transformation on the input while preserving the dimensionality of the original input space. The transcriptome feature is processed in a similar vein. The molecular feature is then concatenated with the sum of the outputs from the two linear layers. This integrated feature representation is subsequently fed into a succession of fully connected layers, with a dimensional hierarchy of [768 + 512, 512, 256, 128, 1]. The ReLU activation function is applied across these layers. The ultimate output is a prediction of the natural logarithm of the half-maximal inhibitory concentration (LN IC50). To train the DL framework, the MSE is employed as the loss function for its proven efficiency in regression problems.

### Validate the model on GDSC and CCLE

#### Dataset

In this study, we used two datasets, GDSC and CCLE, to evaluate the performance of DIPK, respectively. Both datasets provide cell-drug pairs and corresponding IC50, with cell lines providing transcriptome data and drugs providing names. Using the drug names, we accessed the specific molecular structures from PubChem. According to the transcriptome data, we selected top 256 genes with the highest expression levels (see Supplementary Text S2). We extracted these highly expressed genes’ feature from BIONIC and averaged them as the interaction feature. Following standardized data preprocessing and cleaning procedures (see Supplementary Text S1), the GDSC dataset yielded 957 cell lines and 206 drugs, resulting in 159 114 cell-drug pairs. Similarly, the CCLE dataset comprised 550 cell lines and 173 drugs, producing a total of 80 056 cell line-drug pairs.

#### Model training and validation

To validate the model on learned cell lines and drugs with the GDSC dataset, we performed 5-fold cross-validation. The predictions on the 5-fold were averaged to generate more accurate predictions. For baseline Precily, we used the hyperparameters recommended. For validation on unlearned cell lines and drugs with the GDSC dataset, there were no duplicate cell lines or drugs between the training and test sets when splitting the dataset. To make the results statistically significant and to make the training set size as large as possible [25], a 25-fold cross-validation was used. For validation on unlearned cell lines with the CCLE dataset, there were no duplicate cell lines between the training and test sets when splitting the dataset to ensure comparability to the baseline reported. A 5-fold cross-validation was implemented, with the predictions from the 5-fold subsequently averaged. The details are shown in Supplementary Text S3.

### Validate the model with single-cell data

We procured the single-cell RNA sequencing (scRNA-seq) data from the study conducted by Kinker *et al*. through the Gene Expression Omnibus (GEO) with GSE157220 [45]. Due to the absence of IC50, the single-cell dataset consisted of 116 cell lines and 173 drugs, including 17 279 cell line-drug pairs. Notably, cell lines included in this dataset were excluded from the training set. We then used this dataset to validate the model trained on the CCLE dataset. The details are shown in Supplementary Text S1.

### Validate the model with patient data

The clinical patient gene expression data utilized in this study were procured from the GEO with GSE25055, GSE32646, and GSE20194 [28]. Each sample was annotated as pCR, RD (residual disease) or nCR (non-pCR). We used the model trained on the GDSC dataset (to maintain the consistency of expression profile types) to predict drug response to paclitaxel in these different patients. The details are shown in Supplementary Text S1.

## RESULTS

### DIPK improved prediction accuracy and stability

**Figure 2:**
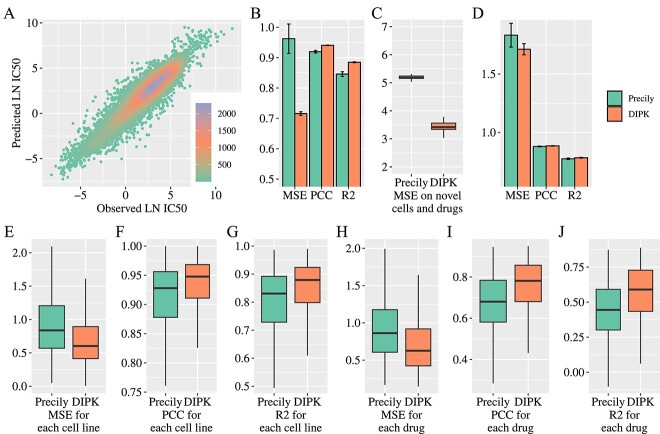
Performance on bulk data. (**A**) Scatter diagram of observed LN IC50 and predicted LN IC50. Density was presented by the difference in color. (**B**) Comparison of the model performance on learned cell lines and drugs with the GDSC dataset. (**C**) Comparison of the model performance on novel cell lines and drugs with the GDSC dataset. (**D**) Comparison of the model performance on the CCLE dataset. (**E**–**G**) Comparison of the model performance for each cell line. (**H**–**J**) Comparison of the model performance for each drug.

The GDSC is a comprehensive resource that provides large-scale pharmacogenomic data to support cancer research [11]. It comprises the gene expression profiling of cancer cell lines analyzed by array technology, along with their corresponding responses (IC50) to various drug compounds [46, 47]. In this study, we evaluated the performance of the model for drug response prediction on the GDSC dataset (both drug response data and gene expression data were obtained from GDSC) using a 5-fold cross-validation. To ensure accurate reporting of the model’s predictive capabilities, we followed a commonly adopted practice of averaging predictions obtained from each of the 5-fold [48]. Figure 2A demonstrates a strong and consistent correlation between the predicted and observed LN IC50 values. To highlight the advantages of DIPK, we compared its performance with that of Precily [29], a recently developed deep neural network-based framework for drug response prediction based on pathway enrichment scores and drug descriptors. As shown in Figure 2B, DIPK outperformed Precily in terms of mean square error (MSE) while also exhibiting higher Pearson correlation coefficient (PCC) and R2 values (DIPK: MSE = 0.7159 $\pm $ 0.0060, PCC = 0.9406 $\pm $ 0.0008, R2 = 0.8848 $\pm $ 0.0015; Precily: MSE = 0.9625 $\pm $ 0.0483, PCC = 0.9198 $\pm $ 0.0043, R2 = 0.8460 $\pm $ 0.0077). For the standard deviation of these indicators, DIPK is lower than Precily, showing strong stability. Furthermore, we conducted an in-depth analysis of the prediction performance for individual cell lines and drugs. As shown in Figure 2E–J, DIPK outperform DIPK across different cell lines and drugs in terms of MSE, correlation and variation, indicating that DIPK achieved enhanced prediction accuracy and improved stability. In contrast to DIPK, Precily employed pathway enrichment scores as a method for characterizing cell lines. The pathway enrichment score serves as a statistical metric indicating the extent to which a specific pathway is enriched with differentially expressed or regulated genes, albeit without considering gene interaction information. The augmentation of gene interaction data in DIPK resulted in enhanced prediction accuracy and stability in drug response forecasting, underscoring the utility of incorporating comprehensive gene interaction information for refined characterization of cell lines in the context of drug response prediction.

In the rapidly evolving landscape of clinical practice, the frequent emergence of new cell lines and drugs presents a formidable challenge in therapeutic decision-making, particularly due to the absence of extant drug response data [49, 50]. The capability to predict drug response for unseen cell lines and new pharmaceutical compounds could serve as a pivotal aid in optimizing therapy [51, 52]. To assess the generalizability of our model in such scenarios, we conducted experiments involving cell lines and drugs that were previously unencountered by the model using the GDSC dataset. As shown in Figure 2C, DIPK has a significantly lower median MSE than Precily, which demonstrates that DIPK has a stronger generalization ability when encountering cell lines and drugs never seen before. This enhanced generalizability can be attributed to the integration of prior knowledge into our model, which enhances the model’s capacity to adapt and make accurate predictions in novel situations.

Gene expression analysis encompasses various techniques with distinct strengths and limitations [53]. The study above relied on gene expression profiles acquired through transcription profiling using array technology [54, 55]. However, it is important to consider alternative techniques to evaluate the model’s effectiveness. RNA sequencing (RNA-seq) has emerged as a widely used high-throughput sequencing technology for gene expression analysis in diverse research fields [56, 57]. Compared to array technology, RNA-seq offers improved accuracy and sensitivity in expression values [58]. The CCLE serves as a public database, providing RNA-seq gene expression profiles of a broad spectrum of cell lines [12]. Here, we employed the CCLE dataset (drug response data were obtained from GDSC and gene expression data were obtained from CCLE) for both training and evaluation of the model. Our experiment simulated the scenario of targeting novel cell lines with existing drugs, ensuring no overlapping cell lines in the training, validation, and testing datasets. We conducted a 5-fold cross-validation and averaged predictions to obtain robust results. For benchmarking, we compared the performance of DIPK with Precily. As shown in Figure 2D, DIPK showed better accuracy and stability in terms of MSE, PCC and R2 (DIPK: MSE = 1.7123 $\pm $ 0.0479, PCC = 0.8849 $\pm $ 0.0018, R2 = 0.7830 $\pm $ 0.0031; Precily: MSE = 1.8327 $\pm $ 0.1016, PCC = 0.8797 $\pm $ 0.0034, R2 = 0.7739 $\pm $ 0.0059), which indicated that DIPK could handle different gene expression profiles obtained by different transcriptome analysis technologies. Unlike the validation results on the GDSC dataset,the comparative advantage of DIPK over Precily on the CCLE dataset appears less conspicuous. This disparity arises from the expanded integration of transcriptome information by Precily. Concretely, Precily delineates cell lines by computing a pathway enrichment score from a comprehensive expression profile that encompasses 17 420 genes for GDSC and 57 820 genes for CCLE. In contrast, DIPK employs a method wherein gene subsets are selectively chosen and encoded into latent vectors, thereby mitigating sensitivity to the total number of genes within the expression profile.

### Drug response prediction using single-cell expression profiles

**Figure 3:**
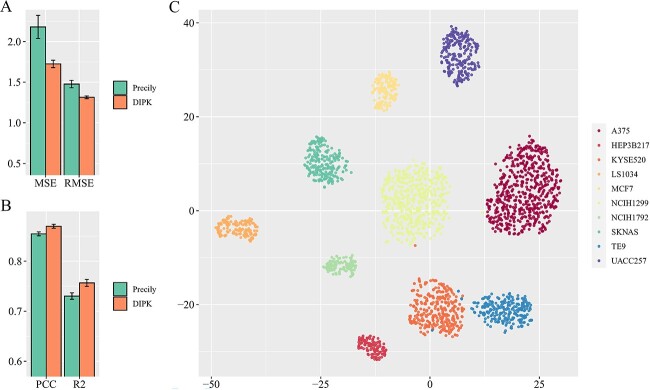
Performance on single-cell expression profiles. (**A**) Comparison of the MSE and RMSE on the test set. (**B**) Comparison of the PCC and R2 on the test set. (**C**) The t-SNE plot showing cell features obtained by DIPK of single cells belonging to 10 random cell lines.

In recent years, the utilization of scRNA-seq has witnessed a remarkable surge owing to its ability to capture the gene expression profiles of single cells [59, 60]. Unlike bulk RNA-seq data, which provide aggregated gene expression levels across multiple cells in a tissue and may obscure the contributions of specific cell subpopulations [61, 62], scRNA-seq data offer a more detailed and heterogeneous view of cellular responses. To assess the model’s capability to predict drug response at the single-cell level, we utilized scRNA-seq data from the study conducted by Kinker *et al*. [45] to construct our test set, including 207 cell lines and 173 drugs. The training set, on the other hand, was built using bulk RNA-seq data from the CCLE dataset, excluding cell lines that appeared in the test set. We performed 5-fold cross-validation, averaging predictions across the folds. As shown in Figure 3A–B, DIPK achieved better performance compared with Precily in terms of MSE, RMSE, PCC and R2, indicating that DIPK was more effective in predicting drug response on scRNA-seq data after training on bulk RNA-seq data. Notably, when compared with Precily, the observed enhancement in predictive accuracy and stability while validating on scRNA-seq data was notably greater than that on bulk RNA-seq data. This observation underscores the robust generalizability of DIPK, evidencing its potency in confronting varying data structures. This phenomenon is due to the fact that when calculating the interactome feature, the group of genes with the highest expression level is extracted without paying attention to the specific expression data, making DIPK relatively insensitive to the differences in bulk or single-cell data. Furthermore, we randomly selected single cells from 10 different cell lines and extracted cell features with DIPK. According to Figure 3C, cell features learned by DIPK can be used to perform cellular fractionation, which indicates that DIPK learned the intrinsic characteristics of the cells well enough to distinguish them.

### DIPK enable reliable clinical response in patients

**Figure 4:**
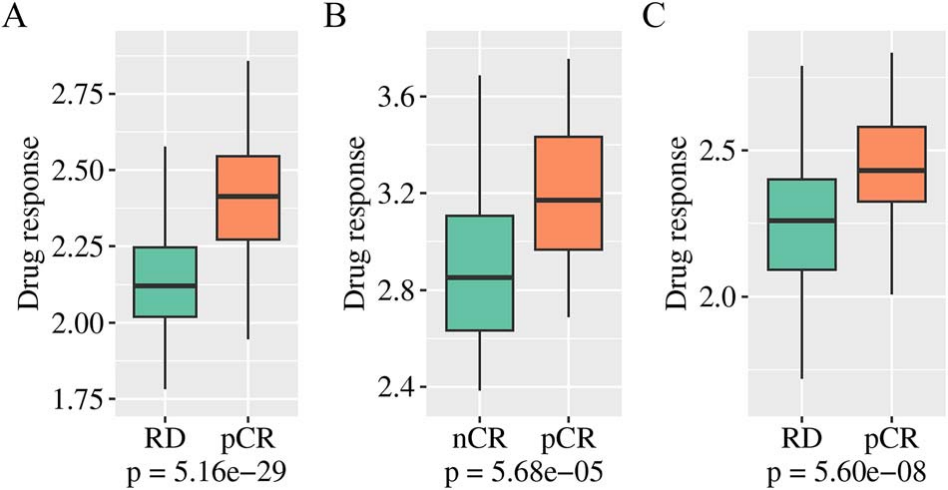
Performance on patient data. (**A**) Comparison of predicted drug response of samples in GSE25055 to paclitaxel between the pCR group and the RD group. (**B**) Comparison of predicted drug response of samples in GSE32646 to paclitaxel between the pCR group and the nCR group. (**C**) Comparison of predicted drug response of samples in GSE20194 to paclitaxel between the pCR group and the RD group. The predicted drug response was measured by predicted -LN IC50. $P$-values were obtained using the two-sided heteroskedasticity *t*-test.

To assess the applicability of DIPK in clinical treatment, we conducted tests using three datasets [28] of breast cancer patients with annotated pCR status to paclitaxel treatment, namely GSE25055, GSE32646 and GSE20194. pCR is defined as the absence of any invasive cancer in breast cancer patients following the completion of neoadjuvant chemotherapy [63, 64]. The samples in each dataset were categorized into two groups based on treatment annotations: the pCR group and the RD or nCR group. Typically, the pCR group exhibits superior drug responses compared to the RD or nCR group. Our objective was to determine whether DIPK could accurately predict these differences. Figure 4A–C illustrates the drug response comparison between the pCR group and the RD or nCR group in all three datasets. The predictions made by DIPK indicated that the drug response of the pCR group was consistently higher than that of the RD or nCR group, confirming that the pCR group indeed displayed a more favorable response to the chemotherapy compound. These findings provide compelling evidence supporting the reliability of DIPK in clinical treatment applications. The ability of DIPK to accurately discern distinct drug response patterns between different patient groups holds significant implications for personalized cancer therapy. It can serve as a valuable tool in guiding treatment decisions, assisting clinicians in selecting the most appropriate therapeutic approaches, and ultimately improving patient outcomes.

### The importance of gene interactome and DAE

To ascertain the individual contributions of each component in DIPK towards its overall performance, we performed a series of ablation experiments. We proposed two auxiliary models, namely the **D**eep neural network integrating prior knowledge of gene **E**xpression and **M**olecular structures (DEM) and the **D**eep neural network integrating prior knowledge of gene **I**nteraction relationships and **M**olecular structures (DIM). DEM is a variant of DIPK from which the interactome features have been excised. DIM represents a version of DIPK devoid of the DAE component. In this framework, the gene expression features are defined by the expression profile without DAE compression. Through this rigorous, comparative investigation of DEM and DIM, we hope to underscore the respective significance of the interactome features and the DAE in DIPK, providing a deeper understanding of the importance of each component in predicting drug response.

**Figure 5:**
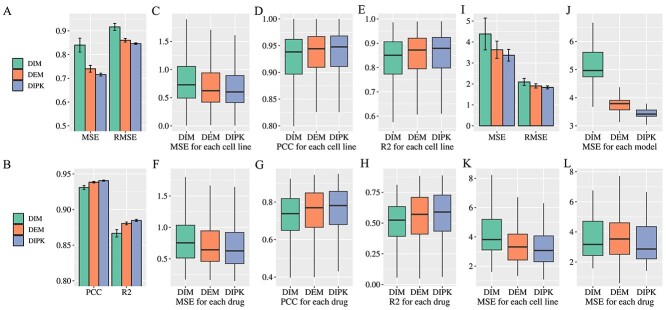
Ablation experiments. (**A**–**B**) Comparison of the model performance on the test set. (**C**–**E**) Comparison of the model performance for each cell line. (**F**–**H**) Comparison of the model performance for each cell drug. (**I**) Comparison of the model performance on the test set. (**J**) Comparison of the model performance on the test set for each fold. (**K**) Comparison of the model performance for each cell line. (**L**) Comparison of the model performance for each drug. (A–H) showed the model performance when validating on learned cell lines and drugs and (I–L) showed the model performance when validating on unlearned cell lines and drugs.

In our pursuit to elucidate the efficacy of integrating the interactome features and the DAE into our model, we conducted the test on learned cell lines and drugs. The evaluation metrics employed in this study were Root Mean Squared Error (RMSE), MSE, PCC and R-squared (R2). In a comparative analysis with DEM and DIM, DIPK consistently exhibited superior performance across all metrics (DIPK: RMSE = 0.8461, MSE = 0.7159, PCC = 0.9406, R2 = 0.8848, DEM: RMSE = 0.8604, MSE = 0.7403, Pearson = 0.9384, R2 = 0.8806, DIM: RMSE = 0.9165, MSE = 0.8399, PCC = 0.9310, R2 = 0.8667), as demonstrated in Figure 5A, B. These results provide robust evidence for the efficacy of the interactome features and the DAE in enhancing the predictive capacity of our model for drug responses. To delve further into the improvements conferred by the interactome features and the DAE, we scrutinized the prediction efficacy for each individual cell line and drug. The findings indicated that DIPK consistently outperforms DEM and DIM across various cell lines and drugs as demonstrated in Figure 5C–H. This denoted that the integration of the interactome features and the DAE not only elevated the accuracy of our model but also enhanced stability, underscoring their utility in the realm of drug response prediction.

Furthermore, we evaluated the impact of incorporating the interactome features and the DAE on novel cell lines and drugs. For a robust assessment, we measured MSE on the test set for each fold in a 25-fold cross-validation procedure as shown in Figure 5J. DIPK demonstrated superior performance in terms of both median value and interquartile range (IQR) compared to DEM and DIM (MSE median $\pm $ IQR: DIPK: 3.4188 $\pm $ 0.2223, DEM: 3.7897 $\pm $ 0.3362, DIM: 4.9669 $\pm $ 0.8720). This observation suggested that the integration of the interactome features and the DAE effectively enhanced the model’s generalization capability. In a subsequent analysis, we calculated the MSE and RMSE when the predicted values across the 25 folds were averaged as shown in Figure 5I. Consistent with earlier observations, DIPK outperformed both DEM and DIM (DIPK: MSE = 3.3705, RMSE = 1.8359, DEM: MSE = 3.6321, RMSE = 1.9058, DIM: MSE = 4.3838, RMSE = 2.0938), further corroborating the enhanced generalization capability of our model. Lastly, we scrutinized the predictive performance of each individual cell line and drug. As shown in Figure 5K, L, DIPK outperforms DEM and DIM, indicating that the interactome feature and DAE effectively enhance the stability, thereby rendering it a potent tool for drug response prediction.

## CONCLUSION AND DISCUSSION

In summary, we presented a DL-based framework named DIPK, which considered the complex interactions of multiple genes in drug response. We demonstrate that DIPK outperforms the existing methods on both GDSC and CCLE datasets in terms of prediction accuracy and stability. Moreover, DIPK exhibits robust generalizability on sc-RNA seq data, indicating its capability to handle diverse data types. Significantly, we demonstrated the reliability of DIPK in clinical treatment analysis. Hence, in clinical practice, DIPK can serve as a tool for screening drugs tailored to specific cancer patient cell lines, enabling the selection of safe and efficacious treatment options. This approach facilitates the realization of personalized and precise medical interventions for patients.

This study elucidates the pivotal role of gene interaction information in computational methods for predicting cellular drug responses, aligning with many studies in the biomedical field that underscore the modulatory influence of concerted and complex interactions among multiple genes on drug responses. Concurrently, empirical investigations across diverse data types underscore the significance of self-supervised pre-training schemes in augmenting model robustness. Notably, this research leveraged genome and transcriptome features for cell line characterization, excluding the incorporation of additional multi-omics data modalities such as proteomics and metabolomics. While the inclusion of multiple omics datasets holds potential for improving model performance—especially with the introduction of specific information on protein targets, enabling more precise drug response predictions for distinct cancer cell types—such augmentation raises concerns regarding model generalization. The reliance on multiple omics data may compromise the model’s applicability when confronted with the absence of a particular omics dataset, such as single-cell or clinical data. In contrast, the gene interaction network information harnessed by DIPK serves as a prior knowledge base, independent of specific data types, ensuring broader applicability across diverse scenarios.

**Key Points**

- Enhanced drug response prediction: the manuscript demonstrates the importance of gene interaction relationships in predicting drug response, and introduces a novel deep learning framework DIPK to integrate this valuable prior knowledge, which outperforms existing methods in predicting drug responses across diverse cell lines and drugs.
- Generalization across diverse data types: we demonstrated the versatility of DIPK in generalizing across diverse data types, including bulk RNA-seq and scRNA-seq. This broad applicability showcases the robustness of DIPK in handling different modalities of data, a crucial aspect in the context of the heterogeneous nature of cancer.
- Reliable clinical applicability: the article underscores the reliable clinical applicability of DIPK through its evaluation of clinical data. The assessment of DIPK’s performance in distinguishing responses to paclitaxel between pCR and RD groups substantiates its clinical relevance, suggesting that DIPK can provide valuable insights for treatment decision-making in clinical practice.

## Supplementary Material

Supplementary_Data_bbae153

## Data Availability

The dataset, source code, trained models and experimental result data are available on GitHub: https://github.com/user15632/DIPK and Google Drive: https://drive.google.com/drive/folders/16hP48-noHi3-c_LP9TcZxkwAzqxgR0VB?usp=sharing.

## Author Biographies

**Pengyong Li** is an assistant professor at Xidian University. He got his Ph.D. at Tsinghua University. His research interests focus on artificial intelligence technologies and drug discovery.

**Zhengxiang Jiang** is an undergraduate at Xidian University. His research interests include deep learning and bioinformatics.

**Tianxiao Liu** received his Bachelor’s degree from Xidian University, and is now a postgraduate at Xidian University. His research interests include deep learning and molecule generation.

**Xinyu Liu** is an assistant professor at Peking University School and Hospital of Stomatology. He received his PhD at Tsinghua University. His research interests include biomaterial and drug design.

**Hui Qiao** is an oncologist at Tai’an Municipal Hospital. His research interests include cancer therapy.

**Xiaojun Yao** received his PhD degree from University Paris 7-Denis Diderot. He works as a professor at Macao Polytechnic University. His current research interests include drug discovery and bioinformatics.
