# Supplementary material for: Improving drug response prediction via integrating gene relationships with deep learning
Source: Brief Bioinform. 2024 Apr 9;25(3):bbae153. doi: 10.1093/bib/bbae153 (PMC11006795; doi:10.1093/bib/bbae153)
Supplement: Supplementary_Data_bbae153 [file supplementary_data_bbae153.docx]

**Improving Drug Response Prediction via Integrating Gene Relationships with Deep Learning**

Pengyong Li,^1,2^ Zhengxiang Jiang,^3^ Tianxiao Liu,^1^ Xinyu Liu,^5^ Hui Qiao^6^ and Xiaojun Yao^7,∗^

1 School of Computer Science and Technology, Xidian University, 710126, Xi’an, Shaanxi, China,

2 State Key Laboratory of Quality Research in Chinese Medicine, Macau Institute for Applied Research in Medicine and Health, Macau University of Science and Technology, 519020, Macau, China,

3 School of Electronic Engineering, Xidian University, 710126, Xi’an, Shaanxi, China,

4 Department of Computer Science, Hunan University, 410082, Changsha, Hunan, China,

5 Beijing Laboratory of Biomedical Materials, Department of Geriatric Dentistry, Peking University School and Hospital of Stomatology, 100081, Beijing, China,

6 Department of Oncology, Tai’an Municipal Hospital, 271021, Tai’an, Shandong, China,

7 Centre for Artificial Intelligence Driven Drug Discovery, Faculty of Applied Sciences, Macao Polytechnic University, 999078, Macao, China

∗ Corresponding author. [xjyao@must.edu.mo](mailto:xjyao@must.edu.mo)

**1 Supplementary Text**

**Text S1: Datasets and preprocessing**

*GDSC:* We leveraged the RMA-normalized microarray basal expression profiles of 1,018 cancer cell lines encompassing 17,737 genes from the GDSC database (https://www.cancerrxgene.org/). To enhance computational efficiency, we narrowed our focus to a subset of genes exhibiting the most substantial variation in expression ^[1]^. After excluding genes with missing expression data, the gene subset consisted of 5,757 genes. PubChem Compound Identifiers (PubChemCIDs) for the drug compounds were obtained from the GDSC database. These identifiers were then used to retrieve the corresponding SMILES strings for the respective compounds, utilizing the Pubchempy library in Python. In cases where PubChemCIDs or SMILES strings for specific drugs were not available through automated means, manual retrieval from the PubChem database (https://pubchem.ncbi.nlm.nih.gov/) and the Library of Integrated Network-based Cellular Signatures (LINCS) (https://lincs.hms.harvard.edu/) was carried out. The observed natural logarithm (LN) transformed IC50 can be obtained from the GDSC database. Finally, the GDSC dataset contains a total of 957 unique cell lines and 206 distinct drugs, yielding 159,114 cell line-drug pairs, and 10% pairs are divided into test sets.

*CCLE:* The RNA sequencing (RNA-seq) data, normalized as Transcripts Per Million (TPM), encompasses 1,019 cancer cell lines and 57,820 genes. It is publicly accessible via the Cancer Cell Line Encyclopedia (CCLE) (https://sites.broadinstitute.org/ccle/) and is utilized as gene expression data of cell lines. Similar to the previous study, we utilized the annotation file to complete the transformation from gene IDs to gene names ^[2]^. Then the gene expression matrix was transformed using the formula log2(TPM+1) ^[3]^. The gene subset selected for analysis consists of 5,863 genes. To transform the preprocessed RNA-seq data into a more comparable format, z-score standardization was performed for each sample. SMILES strings and IC50s are obtained in the same way as in the GDSC section. Finally, the CCLE dataset contains 550 cell lines and 173 drugs, yielding 80,056 cell line-drug pairs, and 10% pairs are divided into test sets.

*Single-cell data:* We procured the Counts Per Million (CPM) normalized single-cell RNA sequencing (scRNA-seq) data from the study conducted by Kinker, G. S. et al through the Broad Institute’s single-cell portal with SCP542, and the Gene Expression Omnibus (GEO) with GSE157220 ^[4]^. The dataset encompasses 207 cell lines and 22,722 genes. We averaged the single-cell expression data at the cell line level and implemented a log2 transformation on the gene expression matrix using the formula log2(CPM+1). Since UMI counts are independent of gene length, this CPM matrix and the TPM matrix above can be considered equivalent ^[5]^. Similar to the CCLE dataset, the test set constructed from the single-cell data covers the interactions of cells with 173 drugs. Finally, the data was z-score standardized. SMILES strings and IC50s are obtained in the same way as in the GDSC section.

*Patient data:* The clinical patient gene expression data utilized in this study were procured from the Gene Expression Omnibus (GEO) with GSE25055, GSE32646, and GSE20194. The normalization of the data was performed using the normalize.quantiles function in the R programming language ^[1]^. Finally, the data was z-score standardized. SMILES strings and IC50s are obtained in the same way as in the GDSC section.

**Text S2: Details of selecting highly expressed genes**

We first analysis the dataset. Specifically, we calculated the average expression intensity of each gene across all cell lines, and then arranged these values in descending order, as shown in Figure S1. Based on differences in the rate of descent of the curve, we can roughly select the expression density of 256th gene as the threshold to divide into high expression and low expression. Moreover, we compared the performances of DIPK use different number of genes including 128,256, and 512 on the GDSC dataset. As shown in Figure S2, the model using 256 genes achieved lowest MSE score.

**Text S3: Details of extracting interaction information from BIONIC**

BIONIC integrate four human gene-gen interaction networks ^[6]^, including the protein communities and disease network, the bioplex network, the interaction network in three quantitative dimensions organized by stoichiometries and abundances, and the proteome-scale map of the human interactome ^[7-10]^. With self-supervised learning, BIONIC employs an autoencoder design to reconstruct each input network by mapping the integrated gene features to a network representation. The optimization process minimizes the disparity between this reconstruction and the original input networks. By prioritizing the fidelity of the network reconstruction, BIONIC compels the learned gene features to encapsulate the maximum interaction information present in the input networks. We extracted the highly expressed genes' features from BIONIC and averaged them as the interaction representation.

**Text S4: Implementation details**

The model was implemented with the PyTorch and PyTorch geometric library. The training and testing processes were conducted with the NVIDIA RTX 3090. DIPK was trained via the standard batch gradient descent method with the error back-propagation algorithm. The Adam optimization algorithm was chosen to update the parameters. We applied a coarse grid search approach over learning rate. As shown in Table S1, the model with learning rate of 1e-05 shows lower MSE than all the others. All the model was trained for 100 epochs with a learning rate of 1e-05 and a batch size of 64. The data, source code, and trained models are available on GitHub: <https://github.com/user15632/DIPK> and Google Drive: <https://drive.google.com/drive/folders/16hP48-noHi3-c_LP9TcZxkwAzqxgR0VB?usp=sharing>.

**2 Supplementary Figure**

**Figure S1 Identification of highly expressed gene number 256**
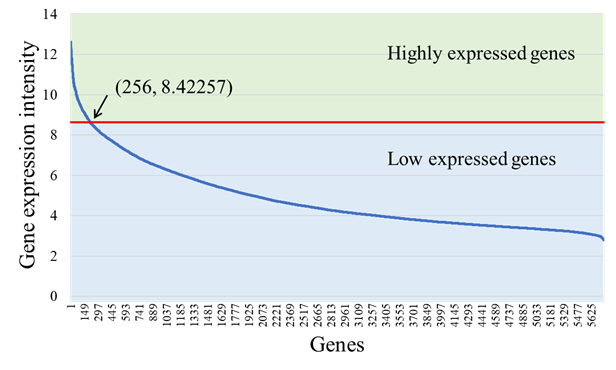


**Figure S2 Performance of different high expression gene numbers**


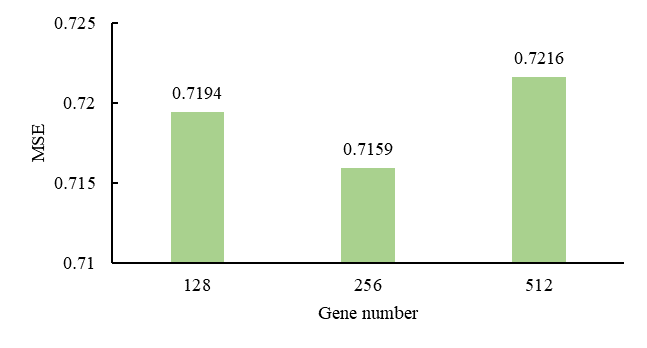


**3 Supplementary Table**

**Table S1: Comparison of MSE with different learning rates**

| Learning rate | MSE |
| --- | --- |
| 1e-03 | 1.5596 |
| 1e-04 | 0.9260 |
| 1e-05 | 0.8576 |
| 1e-06 | 1.3085 |

**Reference**

[1] Jia P, Hu R, Pei G, et al. Deep generative neural network for accurate drug response imputation[J]. Nature communications, 2021, 12(1): 1740.

[2] Chawla S, Rockstroh A, Lehman M, et al. Gene expression based inference of cancer drug sensitivity[J]. Nature communications, 2022, 13(1): 5680.

[3] Tasaki S, Gaiteri C, Mostafavi S, et al. Deep learning decodes the principles of differential gene expression[J]. Nature Machine Intelligence, 2020, 2(7): 376-386.

[4] Kinker G S, Greenwald A C, Tal R, et al. Pan-cancer single-cell RNA-seq identifies recurring programs of cellular heterogeneity[J]. Nature genetics, 2020, 52(11): 1208-1218.

[5] Phipson B, Zappia L, Oshlack A. Gene length and detection bias in single cell RNA sequencing protocols[J]. F1000Research, 2017, 6.

[6] Forster D T, Li S C, Yashiroda Y, et al. BIONIC: biological network integration using convolutions[J]. Nature Methods, 2022, 19(10): 1250-1261.

[7] Huttlin, Edward L., et al. "Architecture of the human interactome defines protein communities and disease networks." Nature 545.7655 (2017): 505-509.

[8] Huttlin, Edward L., et al. "The BioPlex network: a systematic exploration of the human interactome." Cell 162.2 (2015): 425-440.

[9] Hein, Marco Y., et al. "A human interactome in three quantitative dimensions organized by stoichiometries and abundances." Cell 163.3 (2015): 712-723.

[10] Rolland, Thomas, et al. "A proteome-scale map of the human interactome network." Cell 159.5 (2014): 1212-1226.
